# Monoclonal antibodies capable of binding SARS‐CoV‐2 spike protein receptor‐binding motif specifically prevent GM‐CSF induction

**DOI:** 10.1002/JLB.3COVCRA0920-628RR

**Published:** 2021-03-24

**Authors:** Xiaoling Qiang, Shu Zhu, Jianhua Li, Weiqiang Chen, Huan Yang, Ping Wang, Kevin J. Tracey, Haichao Wang

**Affiliations:** ^1^ The Feinstein Institutes for Medical Research Northwell Health Manhasset New York USA; ^2^ Donald and Barbara Zucker School of Medicine at Hofstra/Northwell Hempstead New York USA

**Keywords:** GM‐CSF, cytokine antibody array, surface plasmon resonance, antibody

## Abstract

A severe acute respiratory syndrome (SARS)‐like coronavirus 2 (SARS‐CoV‐2) has recently caused a pandemic COVID‐19 disease that infected approximately 94 million and killed more than 2,000,000 people worldwide. Like the SARS‐CoV, SARS‐CoV‐2 also employs a receptor‐binding motif (RBM) of its envelope spike protein for binding the host angiotensin‐converting enzyme 2 (ACE2) to gain viral entry. Currently, extensive efforts are being made to produce vaccines against a surface fragment of a SARS‐CoV‐2, such as the spike protein, in order to boost protective antibodies that can inhibit virus‐ACE2 interaction to prevent viral entry. It was previously unknown how spike protein‐targeting antibodies would affect innate inflammatory responses to SARS‐CoV‐2 infections. Here we generated a highly purified recombinant protein corresponding to the RBM of SARS‐CoV‐2, and used it to screen for cross‐reactive monoclonal antibodies (mAbs). We found two RBM‐binding mAbs that competitively inhibited its interaction with human ACE2, and specifically blocked the RBM‐induced GM‐CSF secretion in both human peripheral blood mononuclear cells and murine macrophage cultures. Our findings have suggested a possible strategy to prevent SARS‐CoV‐2‐elicited “cytokine storm,” and revealed a potentially anti‐inflammatory and protective mechanism for SARS‐CoV‐2 spike‐based vaccines.

## Graphical Abstract

SARS‐CoV‐2 Spike Protein‐reactive monoclonal antibodies specifically impaired the viral spike protein‐induced GM‐CSF secretion by human peripheral blood mononuclear cells.

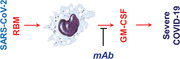

## 1 INTRODUCTION

Shortly after the 2003 outbreak of the severe acute respiratory syndrome (SARS) caused by a β‐coronavirus (SARS‐CoV),^1^ the recent emergence and rapid spread of SARS‐like coronavirus 2, SARS‐CoV‐2, has caused a pandemic COVID‐19 that is catastrophically damaging human health. As of January 19, 2021, approximately 94 million people have been infected, leading to more than 2,000,000 deaths worldwide (https://www.who.int/emergencies/diseases/novel-coronavirus-2019). Like the SARS‐CoV,^1^ SARS‐CoV‐2 virus also employs its envelope spike (S) glycoproteins to recognize and bind a host cell surface receptor, the angiotensin‐converting enzyme 2 (ACE2), to gain host cell membrane fusion and viral entry.^2^, ^3^ Structurally the SARS‐CoV‐2 S protein contains a receptor‐binding domain (RBD) that embraces a receptor‐binding motif (RBM) in a “closed” configuration inaccessible by the host ACE2 receptor. Upon cleavage of the S protein by host proteases such as furin and the transmembrane protease/serine subfamily member 2, the RBD undergoes a conformational change (from a “closed” to an “open” configuration) that enables the “exposure” of RBM to host cell receptors.^3^, ^4^, ^5^, ^6^

In the absence of effective therapies, vaccination has become a key option to boost adaptive antibody responses against SARS‐CoV‐2 infections. One approach is to use a surface fragment of a SARS‐CoV‐2, such as the spike (S) protein as antigens,^7^ in the hope that antibodies targeting the S protein may inhibit viral interaction with host ACE2 receptor to prevent viral entry.^7^ In patients infected by SARS‐CoV or SARS‐CoV‐2, neutralizing antibodies targeting the RBD or RBM region of respective viral S proteins were found^1^, ^2^, ^8^, ^9^, ^10^, ^11^, ^12^, ^13^; and some of them could indeed impair RBD‐ACE2 interaction^14^ and viral entry.^9^, ^12^ Intriguingly, a previous study revealed that antibodies against different epitopes of SARS‐CoV S protein exhibited divergent effects: antibodies targeting RBM (residue 471–503) conferred protection, whereas antibodies targeting epitopes (e.g., residue 597–603) outside of the RBM region adversely worsen the outcomes.^15^ However, it was previously unknown how RBM‐targeting antibodies would affect innate inflammatory responses to SARS‐CoV‐2 infections?

Recently, emerging evidence suggested that ACE2 might also be expressed in human peripheral blood mononuclear cells (hPBMCs)^16^ and murine macrophage‐like RAW 264.7 cells.^16^ Furthermore, hPBMCs produced several proinflammatory cytokines (e.g., TNF, IL‐1β, and IL‐6) and chemokines (e.g., IL‐8 and MIP‐1β) in response to SARS‐CoV S protein stimulation.^17^ However, it was previously unknown how RBM‐binding monoclonal antibodies (mAbs) affect the SARS‐CoV‐2‐elicited innate immune responses. In the present study, we sought to screen for mAbs capable of binding SARS‐CoV‐2 RBM, and determine how these RBM‐binding mAbs affect the RBM‐induced cytokine/chemokine production in hPBMCs and murine macrophage cultures.

## 2 RESULTS AND DISCUSSION

### 2.1 Generation of recombinant RBD and RBM protein fragments of SARS‐CoV‐2

To screen for mAbs capable of binding the RBD or RBM region of SARS‐CoV‐2 spike protein (Supporting Information Fig. S1A), we generated recombinant RBD and RBM corresponding to residue 319–541 and residue 437–508 of SARS‐CoV‐2 spike (S) protein (Supporting Information Fig. S1B). These recombinant proteins were purified from insoluble inclusion bodies by differential centrifugation, urea solubilization, and histidine‐tag affinity chromatography (Supporting Information Fig. S1C). Extensive washings of the immobilized recombinant RBD or RBM proteins with buffer containing 8.0 M urea effectively removed contaminating bacterial endotoxins. Subsequently, the purified RBD and RBM was dialyzed in a buffer supplemented with a reducing agent, Tris (2‐carboxyethyl) phosphine, to prevent excessive oxidation and cross‐linking of the nine and two cysteine (C) residues in RBD and RBM, respectively (Supporting Information Fig. S1B). As shown in Supporting Information Figure S1D, amino acid sequence analysis revealed a high homology between a tyrosine (Y)‐rich segment (YNYLYR) of SARS‐CoV‐2 RBM and the epitope sequence (NDAL *YEYLR* Q) of several mAbs that we recently generated against human tetranectin (TN),^18^ suggesting a possibility that some TN‐binding mAbs might cross‐react with SARS‐CoV‐2.

### 2.2 Recombinant RBM interacted with human ACE2 (hACE2) and some TN‐binding mAbs

**FIGURE 1:**
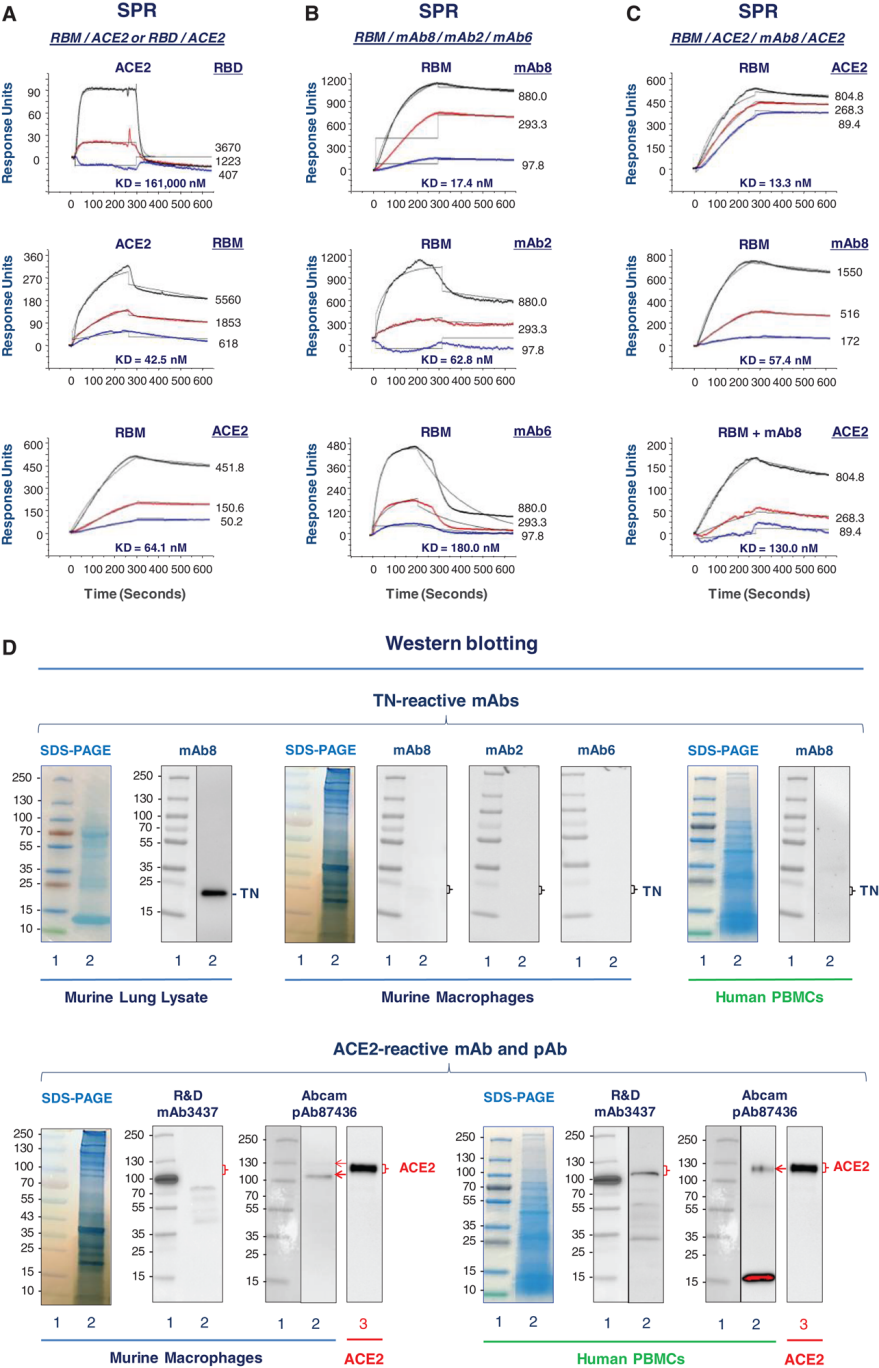
**Receptor‐binding motif (RBM)‐reacting mAbs interferes with RBM‐ACE2 (angiotensin‐converting enzyme 2) interaction**. (**A**) Recombinant severe acute respiratory syndrome (SARS)‐like coronavirus 2 (SARS‐CoV‐2) RBM binds to human angiotensin‐converting enzyme 2 (hACE2) receptor. Highly purified extracellular domain of hACE2 or RBM was immobilized on a sensor chip, and recombinant receptor‐binding domain (RBD), RBM, or ACE2 was applied as an analyte at various concentrations to estimate the dissociation equilibrium constant (*K*_D_). (**B**) RBM cross‐reacted with several monoclonal antibodies (mAbs). Highly purified recombinant RBM was immobilized on the sensor chip, and several human tetranectin (TN)‐reactive mAbs were separately applied as an analyte at various concentrations to assess the *K*_D_ for RBM‐mAb interactions. (**C**) A RBM‐reactive mAb interfered with RBM‐ACE2 interaction. Highly purified recombinant RBM was immobilized on the sensor chip, and recombinant hACE2 was applied as an analyte at various concentrations to assess the *K*_D_ for RBM‐ACE2 interactions. After extensive washing, mAb8 was applied at indicated concentrations, before ACE2 were re‐applied to the RBM‐conjugated sensor chip at identical concentrations (as in top panel). The almost 10‐fold increase in the *K*_D_ (from 13.3 to 130.0 nM) and an almost 3‐fold decrease (from 500 to 165) in the response units suggested that pretreatment with mAb8 markedly inhibited RBM‐ACE2 interaction. (**D**) Western blotting analysis of TN and ACE2 in macrophage and human peripheral blood mononuclear cells (hPBMCs) cultures. Lysates of murine lungs, murine macrophage‐like RAW 264.7 cells, and hPBMCs were resolved on SDS‐PAGE gels, and immunoblotted with various TN‐ or ACE2‐reactive antibodies. CHO cell‐derived and purified recombinant hACE2 (Gln18‐Ser740) containing a Carboxyl TG‐8×Histidine‐GGQ tag was used as a positive control for ACE2‐specific Western blotting analysis

To evaluate the ACE2‐binding properties of recombinant RBD or RBM, the extracellular domain of hACE2 was immobilized to the nitrilotriacetic acid (NTA) sensor chip, and recombinant RBD or RBM was applied as analytes at different concentrations to estimate the dissociation equilibrium constant (*K*_D_) using the Open SPR technique. Surprisingly, our recombinant RBD exhibited an extremely low affinity to the extracellular domain of hACE2 (Fig. 1A, upper panel), with an estimated *K*_D_ of 161,000 nM. It was postulated that the cysteine‐rich RBD was not likely refolded into a “correct” conformation suitable for RBM‐ACE2 interaction, because the high probability of “incorrect” disulfide cross‐linking was factorially proportional to its high number of cysteine residues. In contrast, the *K*_D_ for ACE2‐RBM interaction ranged around 42.5–64.1 nM, regardless whether ACE2 (Fig. 1A, middle panel) or RBM (Fig 1A, bottom panel) was conjugated to the NTA sensor chip before respective application of RBM or ACE2 as analytes at different concentrations. Given the proximity between our estimated *K*_D_ for RBM‐ACE2 interaction and the previously reported *K*_D_ (15–44.2 nM) for SARS‐CoV‐2 S protein‐ACE2 interaction,^5^, ^19^ we concluded that the ACE2‐binding property was at least partly preserved in our recombinant RBM.

Therefore, we conjugated a highly purified recombinant RBM on an NTA sensor chip, and use it to screen for SARS‐CoV‐2 RBM‐binding mAbs. In agreement with a homology between SARS‐CoV‐2 RBM and epitope sequence of several TN‐specific mAbs (Supporting Information Fig. S1D), we found that two (e.g., mAb8 and mAb2) out of three mAbs (i.e., mAb8, mAb2, and mAb6) capable of recognizing a homologous epitope sequence (NDALYEYLRQ)^18^ exhibited a dose‐dependent interaction with RBM (Fig. 1B, top and middle panel), with an estimated *K*_D_ of 17.4 and 62.8 nM, respectively. This estimated *K*_D_ was comparable to that of other SARS‐CoV‐2 RBD‐binding neutralizing antibodies (*K*_D_ = 14–17 nM) recently isolated from COVID‐19 patients.^20^, ^21^ In contrast, another TN‐reactive mAb (mAb6) exhibited an almost 10‐fold lower affinity (as judged by the difference of *K*_D_ between 17.4 nM and 180 nM) and a 2.5‐fold lower response (as judged by the difference of response units between 450 nM and 1125 nM) to RBM as compared with mAb8 (Fig. 1B, bottom panel). Amino acid sequence analysis of the complementarity‐determining regions (CDR) of these three different mAbs (mAb8, mAb2, and mAb6) revealed the presence of two distinct residues (Y and R) in the CDR1 and CDR2 of mAb6 (Supporting Information Fig. S1E), which might underlie its relatively weaker affinity to RBM as compared with other two homologous mAbs (mAb8 and mAb2, Supporting Information Fig. S1E).

### 2.3 RBM‐binding mAbs competitively inhibited RBM‐ACE2 interaction

We then tested whether pretreatment of RBM‐conjugated sensor chip with RBM‐binding mAb competitively inhibited subsequent RBM‐ACE2 interactions. When conjugated to a sensor chip, the recombinant RBM exhibited a dose‐dependent interaction with the extracellular domain of hACE2 (Fig. 1C, top panel), as well as a RBM‐binding mAb (mAb8) (Fig. 1C, middle panel). However, after pretreatment with mAb8, the maximal response unit was markedly reduced from ∼500 (Fig. 1C, top panel) to 175 (Fig. 1C, bottom panel) when ACE2 was applied as an analyte to the RBM‐coated sensor chip at identical concentrations. Meanwhile, the estimated *K*_D_ for RBM‐ACE2 interaction was increased by an almost 10‐fold from 13.3 nM (Fig. 1C, top panel) to 130.0 nM (Fig. 1C, bottom panel), suggesting that RBM‐binding mAbs competitively inhibited RBM‐ACE2 interactions.

Although these two TN‐specific mAbs (mAb8 and mAb2) cross‐reacted with RBM of SARS‐CoV‐2, they specifically reacted with a single band matching the projected molecular weight (MW) of TN in human and mouse serum,^18^ as well as in the murine lung tissue (Fig. 1D) known to express TN.^18^ In contrast, these TN/RBM‐reactive mAbs did not cross‐react with any other proteins in the whole‐cell lysate of murine macrophages (Fig. 1D) or hPBMCs (Fig. 1D), confirming a lack of cross‐reactivity to any other endogenous proteins of innate immune cells. Meanwhile, we also assessed the expression level of ACE2 in murine macrophages and hPBMCs using the R&D rat mAb#3437 as well as the Abcam rabbit pAb #ab87436 recently employed to measure ACE2 expression by flow cytometry.^16^ Although mAb#3437 did not cross‐react with any proteins within the projected MW range (110–135 kDa) of ACE2 in murine macrophage cultures (Fig. 1D, left panels), it did recognize a sharp band matching the MW of ACE2 in hPBMCs (Fig, 1D, right panels). Consistent with a recent report,^16^ we found that the Abcam (Cambridge, MA, USA) rabbit pAb#ab87436 indeed reacted with a purified recombinant hACE2 (Fig. 1D), and recognized two distinct bands within the projected MW range of ACE2 in murine macrophage cultures (Fig. 1D). These two bands might be indicative of distinct forms of murine ACE2 with different degree of post‐translational modification (e.g., glycosylation). Furthermore, this pAb#ab87436 also recognized a band with a MW similar to hACE2 (Fig, 1D), confirming a recent notion that ACE2 is positively expressed in innate immune cells.^16^

### 2.4 RBM‐binding mAbs specifically blocked the RBM‐induced GM‐CSF secretion in primary hPBMCs

**FIGURE 2:**
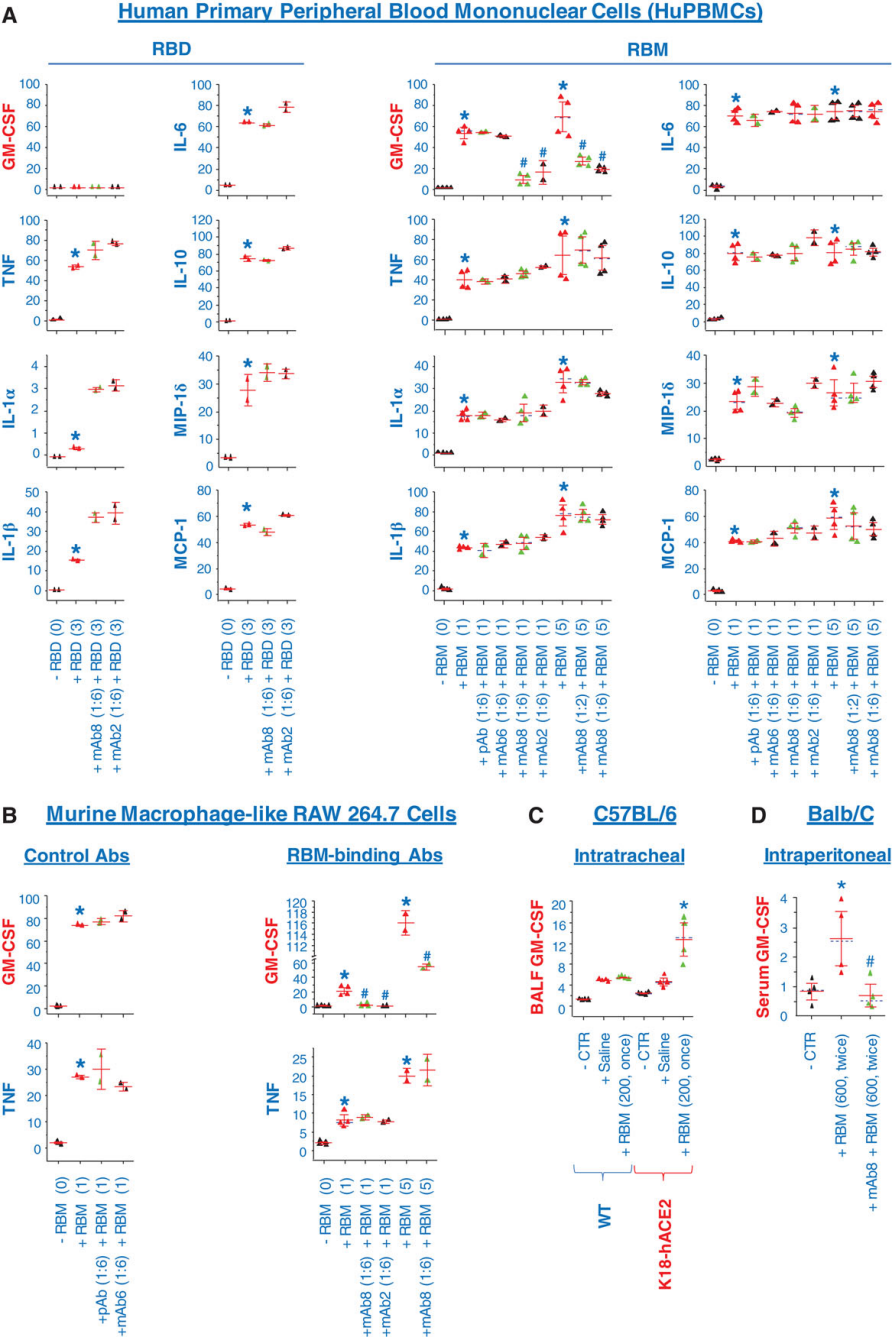
**Receptor‐binding motif (RBM)‐reactive monoclonal antibodies (mAbs) specifically abrogated the RBM‐induced secretion of GM‐CSF in human peripheral blood mononuclear cells (hPBMCs) and murine macrophages**. (**A**) RBM‐reactive mAbs selectively inhibited RBM‐induced GM‐CSF secretion in hPBMCs. The hPBMCs were isolated from blood of healthy donors, and stimulated with recombinant receptor‐binding domain (RBD; 3.0 μg/ml) or RBM (1.0 or 5.0 μg/ml) in the absence or presence of control antibodies (murine polyclonal antibodies, pAb or mAb6) or RBM‐binding mAbs (mAb8 or mAb2, at a molar ratio of 1:2 or 1:6). At 16 h post stimulation, the extracellular concentrations of 42 different cytokines and chemokines were determined by cytokine antibody arrays, and normalized by the positive controls (“+”) on respective membranes. *, *P* < 0.05 vs. negative controls (“− RBM” or “− RBD”); #, *P* < 0.05 vs. positive controls (“+ RBM”) at respective concentrations. (**B**) RBM‐reactive mAbs selectively blocked the RBM‐induced GM‐CSF secretion in murine macrophage‐like RAW 264.7 cells. Murine macrophage‐like RAW 264.7 cells were stimulated with recombinant RBM (1.0 or 5.0 μg/ml) either alone or in the presence of one polyclonal antibodies (pAb) and three different mAbs (mAb6, mAb8, and mAb2; at a molar ratio of 1:6), and extracellular concentrations of 62 cytokines and chemokines were measured by cytokine antibody arrays at 16 h post stimulation. *, *P* < 0.05 vs. negative controls (“− RBM”); #, *P* < 0.05 vs. positive controls (“+ RBM”) at respective concentrations. (**C**) Intratracheal RBM administration elevated bronchoalveolar GM‐CSF in transgenic mice overexpressing human angiotensin‐converting enzyme 2 (ACE2). Male wild‐type B57BL/6 or transgenic mice overexpressing human ACE2 (hACE2) in the epithelial cells (K18‐hACE2 mice) were intratracheally administered with saline (100 μl) or saline containing RBM (200 μg). At 24 h post RBM administration, bronchoalveolar lung fluid was collected and subjected to Cytokine Antibody Assays (*n* = 4). *, *P* < 0.05 vs. saline controls (“+ Saline”). (**D**) RBM‐reactive mAb8 attenuated the RBM‐induced GM‐CSF induction in vivo. Male Balb/C mice were i.p. and repetitively administered with recombinant RBM (at *t* = 0 and *t* = 12 h) at a higher dose (600 μg/mouse) either alone or in combination with a RBM‐binding mAb (mAb8, 2.0 mg/mouse) at the same time. At 16 h post the initial RBM administration, animals were euthanized to harvest blood to measure serum levels of cytokines and chemokines using cytokine antibody arrays (*n* = 4). *, *P* < 0.05 vs. a negative control (“+ Saline”). #, *P* < 0.05 vs. a positive control (“+ RBM” alone)

To examine the possible impact of RBM‐binding mAbs on its immuno‐stimulatory properties, hPBMCs were stimulated with recombinant RBD or RBM in the absence or presence of RBM‐binding mAbs (mAb8 and mAb2), and the levels of 42 different cytokines and chemokines were measured simultaneously by cytokine antibody arrays. In agreement with a previous report that SARS‐CoV spike (S) protein stimulated hPBMCs to produce proinflammatory cytokines (e.g., IL‐1β, IL‐6, and TNF),^17^ we observed a marked elevation of these three cytokines in the RBD‐ or RBM‐stimulated hPBMCs (Supporting Information Fig. S2A; Fig. 2A). In addition, both RBD and RBM also markedly stimulated the secretion of an anti‐inflammatory cytokine (IL‐10) and two chemokines (MIP‐1δ and MCP‐1) in parallel (Supporting Information Fig. S2A; Fig. 2A). Astonishingly, our highly purified RBM, but not RBD, also markedly induced the secretion of a myeloid growth factor, the GM‐CSF in hPBMCs (Supporting Information Fig. S2A; Fig. 2A). However, the co‐addition of two RBM‐binding mAbs (mAb8 and mAb2) similarly and specifically impaired the RBM‐induced secretion of GM‐CSF (Supporting Information Fig. S2A; Fig. 2A) without affecting the RBM‐induced release of other cytokines (e.g., IL‐1β, IL‐6, IL‐10 and TNF) or chemokines (MIP‐1δ and MCP‐1). In contrast, mAbs exhibiting low affinity to RBM (e.g., mAb6) or irrelevant murine polyclonal antibodies (pAbs, IgGs) did not affect the RBM‐induced secretion of GM‐CSF (Supporting Information Fig. S2B, Fig. 2A).

### 2.5 RBM‐binding mAbs also specifically blocked the RBM‐induced GM‐CSF secretion in murine macrophage‐like RAW 264.7 cells

To further confirm the GM‐CSF‐inducing activities of SARS‐CoV‐2 RBM, we stimulated murine macrophage‐like RAW 264.7 cells with highly purified RBM in the absence or presence of RBM‐binding mAbs, and measured the extracellular levels of 62 different cytokines by cytokine antibody arrays. Compared with hPBMCs, murine macrophages appeared to be less responsive to RBM stimulation, and released relatively fewer cytokines (including GM‐CSF and TNF) after stimulation (Supporting Information Fig. S3; Fig. 2B). The mAbs exhibiting low affinity to RBM (e.g., mAb6) or irrelevant murine polyclonal antibodies (pAbs, IgGs) did not affect the RBM‐induced GM‐CSF secretion (Supporting Information Fig. S3A; Fig. 2B). However, two different mAbs exhibiting strong RBM‐binding activities selectively blocked the RBM‐induced GM‐CSF secretion in macrophage cultures without affecting the RBM‐induced TNF secretion (Supporting Information Fig. S3B; Fig. 2B).

It is known that wild‐type mice are generally less susceptible to SARS‐CoV‐2 infections partly because murine ACE2 does not bind to SARS‐CoV‐2 as efficiently as hACE2.^22^ Accordingly, transgenic mice overexpressing hACE2 in epithelial cells under the control of human cytokeratin 18 (K18) promoter, the K18‐hACE2 mice,^23^ have been generated and proven more susceptible to SARS‐CoV‐2 infection, manifested by more exaggerated lung inflammation and injury following intranasal virus inoculation.^23^, ^24^ Consistently, we found that lung ACE2 levels were elevated by 1–2 folds in K18‐hACE2 mice as compared with gender‐ and age‐matched wild‐type C57BL/6 controls (Supporting Information Fig. S4A). It is possible that these two cross‐reactive bands correspond to distinct forms of ACE2 with different degree of post‐translational modification (e.g., glycosylation). Furthermore, intratracheal administration of recombinant RBM (200 μg/mouse) did not obviously increase bronchoalveolar GM‐CSF levels in the wild‐type C57BL/6 mice (Supporting Information Fig. S4B; Fig. 2C), but induced a 2‐3‐fold elevation of bronchoalveolar GM‐CSF levels in two out of four K18‐hACE2 mice (Supporting Information Fig. S4B; Fig. 2C).

Although wild‐type mice are less susceptible to SARS‐CoV‐2 infections,^22^ repetitive administration of recombinant RBM at extremely higher doses (600 μg/mouse) also led to a slight but significant increase of blood GM‐CSF levels, which was similarly reduced by the co‐administration of a RBM‐neutralizing mAb8 (Supporting Information Fig. S4C; Fig. 2D). Our findings fully support the emerging notion that GM‐CSF might be a key feature of SARS‐CoV‐2‐induced cytokine storm in COVID‐19 patients,^25^, ^26^ and suggest an exciting possibility to attenuate the SARS‐CoV‐2‐induced GM‐CSF production and “cytokine storm” in clinical settings using vaccines capable of eliciting RBM‐targeting antibodies (Supporting Information Fig. S5).

“Cytokine storm” refers a hyperactive inflammatory response manifested by the excessive infiltration, expansion, and activation of myeloid cells (e.g., monocytes and macrophages) and consequent production of various cytokines and chemokines (e.g., GM‐CSF, TNF, IL‐1β, IL‐6, and MCP‐1).^27^, ^28^ It has also been suggested as a “driver” of the disease progression particularly in a subset (∼ 20%) of COVID‐19 patients with more severe pneumonia that often escalates to respiratory failure and death.^27^, ^28^, ^29^, ^30^ Furthermore, GM‐CSF might also be a key mediator of the cytokine storm in COVID‐19 and other inflammatory diseases.^31^, ^32^ First, GM‐CSF was up‐regulated before TNF, IL‐6, and MCP‐1 in animal model of SARS‐CoV infection,^33^ and its excessive production adversely contributed to the SARS‐CoV‐induced lung injury.^33^ Second, consistent with the critical contribution of myeloid cells to cytokine storm,^29^ the percentage of GM‐CSF‐expressing leukocytes was significantly increased in a subset of patients with severe COVID‐19.^34^, ^35^ Thus, the excessive production of GM‐CSF may adversely propagate a dysregulated cytokine storm in a subset of COVID‐19 patients (Supporting Information Fig. S5). On the one hand, GM‐CSF can promote myelopoiesis by mobilizing progenitor myeloid cells to sites of SARS‐CoV‐2 infection, and facilitating their proliferation and differentiation into various innate immune cells, such as monocytes, macrophages, and dendritic cells.^31^ On the other hand, GM‐CSF can also polarize mature myeloid cells into a proinflammatory phenotype, promoting the production of various proinflammatory cytokines (e.g., TNF, IL‐1β, and IL‐6) and chemokines (e.g., MCP‐1).^31^

Currently, GM‐CSF has attracted substantial interest as a therapeutic target for the clinical management of COVID‐19.^32^, ^36^ For instance, several companies were actively planning for COVID‐19 clinical trials using mAbs against GM‐CSF (Clinical Trial Registry #: NCT04341116, NCT04351243, NCT04351152, NCT04376684)^32^, ^36^, ^37^ or GM‐CSF receptor.^38^ It has recently been shown that repetitive i.v. infusion of an anti‐human GM‐CSF mAb (Lenzilumab, 600 mg, thrice) significantly improved blood oxygenation, and simultaneously reduced blood levels of two proinflammatory cytokines (e.g., IL‐1α and IL‐6) in 11 out of 12 patients with severe COVID‐19.^39^ Similarly, a neutralizing antibody against human GM‐CSF receptor (mavrilimumab) significantly improved clinical outcome in 13 patients with severe COVID‐19 pneumonia as compared with a cohort of COVID‐19 patients subjected to standard clinical management.^38^ It will thus be important to verify whether our GM‐CSF‐inhibiting mAbs (mAb8 or mAb2) are similarly protective against SARS‐CoV‐2 infection in experimental and clinical settings. This is relevant because our mAbs have already been proven protective in animal model of sepsis partly by reversing immunosuppression and enhancing antimicrobial immune responses.^18^ In light of the ongoing effort in developing effective SARS‐CoV‐2 vaccines, it may be important to assess the innate immune‐modulating properties of all vaccine candidates and respective antibodies in experimental and clinical settings.^40^

This concise report has several obvious limitations. (i) The intricate molecular mechanisms underlying the regulation of RBM‐induced GM‐CSF production were not investigated in the present study. (ii) It is presently not yet known whether genetically silencing ACE2 would abrogate the RBM‐induced GM‐CSF production. (iii) It remains elusive whether some SARS‐CoV‐2‐elicited antibodies also cross‐react with host proteins in such a way that may adversely compromise the half‐life and/or efficacy of some protective antibodies. Nevertheless, our present study has suggested a novel strategy to prevent SARS‐CoV‐2‐elicited “cytokine storm” using RBM‐targeting antibodies, and revealed a potentially anti‐inflammatory and protective mechanism for SARS‐CoV‐2 spike‐based vaccines. It also provided an experimental reagent (RBM) and immune cell‐based assay for the ongoing investigation of the complex pathophysiology of COVID‐19 as well as evaluation of the protective efficacy and innate immune‐modulating properties of various SARS‐CoV‐2 vaccines.

## DISCLOSURES

H.W., J.L., W.C., and K.J.T. are co‐inventors of a patent application (“Tetranectin‐targeting monoclonal antibodies to fight against lethal sepsis and other pathologies”). H.W., K.J.T., J.L., X.Q., and S.Z. are co‐inventors of a provisional patent application (“Use of SARS‐CoV‐2 receptor binding motif (RBM)‐reactive monoclonal antibodies to treat COVID‐19″). All other authors declare no conflicts of interest.

## AUTHORSHIP

X.Q. performed the innate immune cell‐based experiments; S.Z. performed the Open SPR experiments; W.C. and H.Y. performed the in vivo animal experiments; J.L. and K.J.T. generated the recombinant RBM and RBD proteins; P.W. provided important inputs to the experimental design; and H.W. supervised the study, interpreted the results, and wrote the manuscript.

Xiaoling Qiang, Shu Zhu, Jianhua Li, and Weiqiang Chen contributed equally.

## Supporting information

Figure S1. Generation of the ACE2 receptor‐binding domain (RBD) and receptor‐binding motif (RBM) of SARS‐CoV‐2 spike protein. A) Schematic diagram of SARS‐CoV spike protein (S) and its ACE2 receptor binding domain (RBD) and motif (RBM).

Figure S2. RBM‐reactive mAbs specifically abrogated the RBM‐induced secretion of GM‐CSF in human peripheral blood mononuclear cells (hPBMCs).

Figure S3. RBM‐reactive mAbs blocked the RBM‐induced GM‐CSF secretion in murine macrophage‐like RAW 264.7 cells.

Figure S4. RBM induced GM‐CSF production in vivo. A) Western blotting analysis of lung ACE2 levels.

Figure S5. Proposed model for mAb‐mediated inhibition of SARS‐CoV‐2 RBM‐induced GM‐CSF secretion.

Supplementary Material
