# Supplementary material for: Monoclonal antibodies capable of binding SARS‐CoV‐2 spike protein receptor‐binding motif specifically prevent GM‐CSF induction
Source: J Leukoc Biol. 2021 Mar 24;111(1):261–7. doi: 10.1002/JLB.3COVCRA0920-628RR (PMC8251270; doi:10.1002/JLB.3COVCRA0920-628RR)
Supplement: Supplementary file 1 — Figure S1. Generation of the ACE2 receptor‐binding domain (RBD) and receptor‐binding motif (RBM) of SARS‐CoV‐2 spike protein. A) Schematic diagram of SARS‐CoV spike protein (S) and its ACE2 receptor binding domain (RBD) and motif (RBM). [file JLB-111-261-s002.pdf]

Diagram illustrating the hierarchical structure of the SARS-CoV-2 spike protein. The process starts with the **Coronavirus (CoV)**, which contains the **Spike (S) trimer**. This trimer is composed of **S monomer** units. Each S monomer contains the **Receptor-binding domain (RBD)**. The RBD is further detailed as containing the **Receptor-binding motif (RBM)**.

[illegible]

```

449  YNLYLYR  454      (SASR-CoV-2)
      | : | |
NDALYEYL-RQ      (human TN)
| : | : | : | :
NEALFEYA-RH      (murine TN)

```

mAb#	CDR1-H	CDR2-H	CDR3-H	KD hTN	KD mTN	KD RBM
mAb8	TDXXX-----XXXXXXXXXX	TXXXXXXXXXX	XVXXXXLXX	2.0e-9	1.5e-8	1.7e-8
mAb2	SDXXX-----XXXXXXXXXX	TXXXXXXXXXX	XGXXLMXX	1.1e-10	2.3e-8	6.8e-8
mAb6	SYXXX-----XXXXXXXXXX	RXXXXXXXXXX	XGXXLXX	9.2e-10	1.0e-7	1.8e-7

	CDR1-L	CDR2-L	CDR3-L			
mAb8	XXXXXXXXXXYXX-----KXXXXXX-----XXNXXXXX	2.0e-9	1.5e-8	1.7e-8		
mAb2	XXXXXXXXXXHXX-----YXXXXXX-----XXKXXXXXX	1.1e-10	2.3e-8	6.8e-8		
mAb6	XXXXXXXXXXHXX-----YXXXXXX-----XXKXXXXXX	9.2e-10	1.0e-7	1.8e-7		

Figure S1. Generation of the ACE2 receptor-binding domain (RBD) and receptor-binding motif (RBM) of SARS-CoV-2 spike protein. **A)** Schematic diagram of SARS-CoV spike protein (S) and its ACE2 receptor binding domain (RBD) and motif (RBM). **B)** Amino acid sequence of RBD and RBM of SARS-CoV and SARS-CoV-2. RBM sequence is denoted by text in green; “@”, denote residues in close contact with ACE2. **C)** SARS-CoV-2 spike protein RBD and RBM corresponding to amino acids 319-541 and 437-508 with an N-terminal histidine tag were expressed in *E. coli* BL21 (DE3) pLysS cells, and purified by differential centrifugation of inclusion bodies, urea solubilization and histidine-tag affinity chromatography. **D)** SARS-CoV-2 RBM contains a sequence highly homologous to the epitope sequence of human and murine TN for several anti-TN monoclonal antibodies (mAbs). **E)** Comparison of the complementarity-determining regions (CDRs) of heavy (“H”) and light (“L”) chains of three different mAbs. “X”, denotes identical amino acid residues among three different mAbs (mAb8, mAb2, and mAb6). “-“, denotes amino acid residues of the linkers between different CDRs. K_D , the equilibrium dissociation constant of three mAbs for three different antigens (human TN, murine TN, and RBM, respectively).
