# Supplementary material for: Monoclonal antibodies capable of binding SARS‐CoV‐2 spike protein receptor‐binding motif specifically prevent GM‐CSF induction
Source: J Leukoc Biol. 2021 Mar 24;111(1):261–7. doi: 10.1002/JLB.3COVCRA0920-628RR (PMC8251270; doi:10.1002/JLB.3COVCRA0920-628RR)
Supplement: Supplementary file 2 — Figure S2. RBM‐reactive mAbs specifically abrogated the RBM‐induced secretion of GM‐CSF in human peripheral blood mononuclear cells (hPBMCs). [file JLB-111-261-s005.pdf]

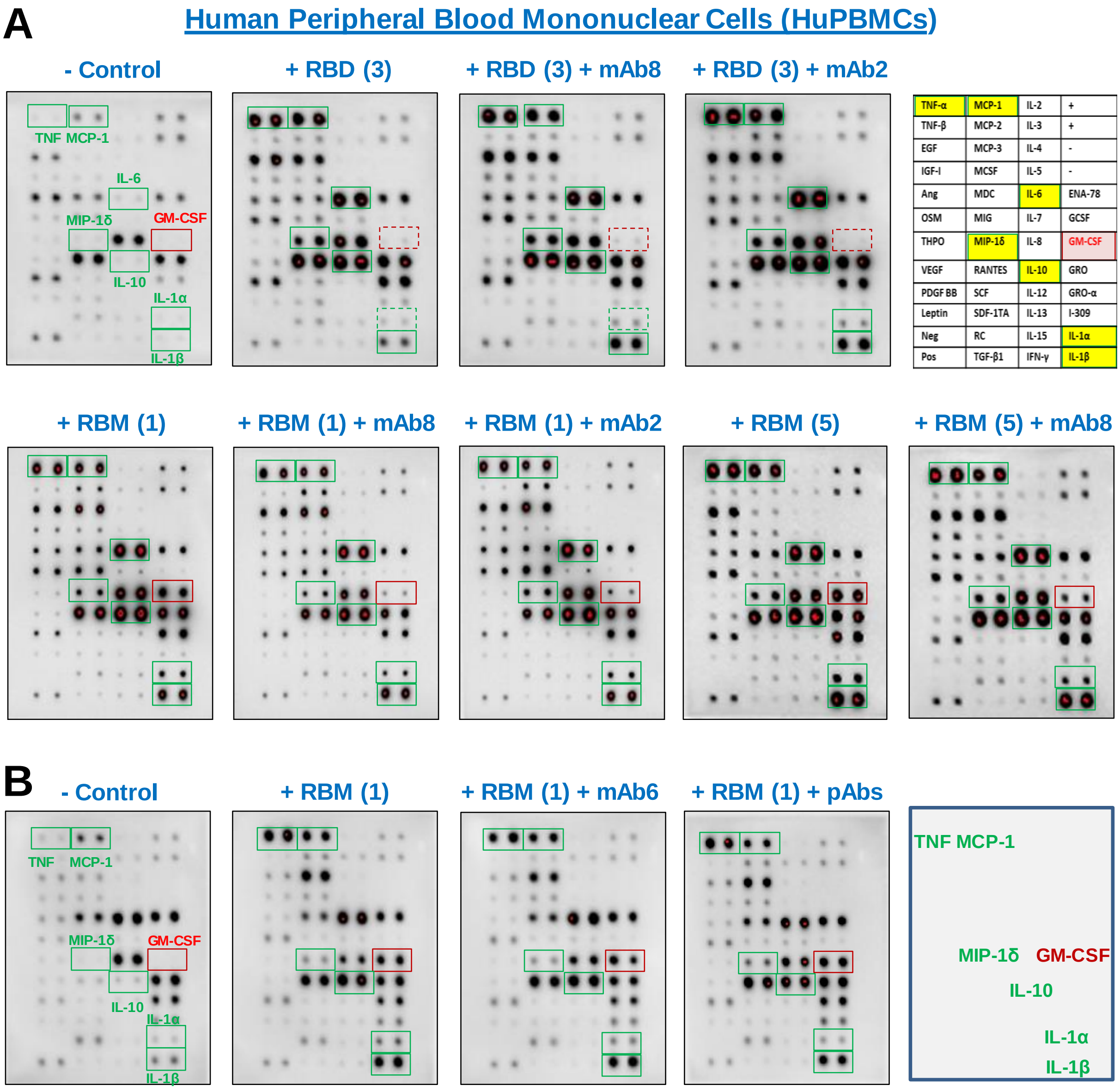

Figure S2. RBM-reactive mAbs specifically abrogated the RBM-induced secretion of GM-CSF in human peripheral blood mononuclear cells (hPBMCs). Human peripheral blood mononuclear cells (hPBMCs) were isolated from blood of healthy donors, and stimulated with recombinant RBD (3.0 µg/ml) or RBM (1.0 or 5.0 µg/ml) in the absence or presence of RBM-binding mAbs (mAb8 or mAb2, at a molar ratio of 1:2 or 1:6) or irrelevant murine polyclonal antibodies (pAbs). At 16 h post stimulation, the extracellular concentrations of 42 different cytokines and chemokines were determined by Cytokine Antibody Arrays, and normalized by the positive controls ("+") on respective membranes (Panel B). Shown here were some representative human Cytokine Antibody Arrays included in the quantitative bar graph of Figure 2A.
