# Supplementary material for: Monoclonal antibodies capable of binding SARS‐CoV‐2 spike protein receptor‐binding motif specifically prevent GM‐CSF induction
Source: J Leukoc Biol. 2021 Mar 24;111(1):261–7. doi: 10.1002/JLB.3COVCRA0920-628RR (PMC8251270; doi:10.1002/JLB.3COVCRA0920-628RR)
Supplement: Supplementary file 3 — Figure S3. RBM‐reactive mAbs blocked the RBM‐induced GM‐CSF secretion in murine macrophage‐like RAW 264.7 cells. [file JLB-111-261-s006.pdf]

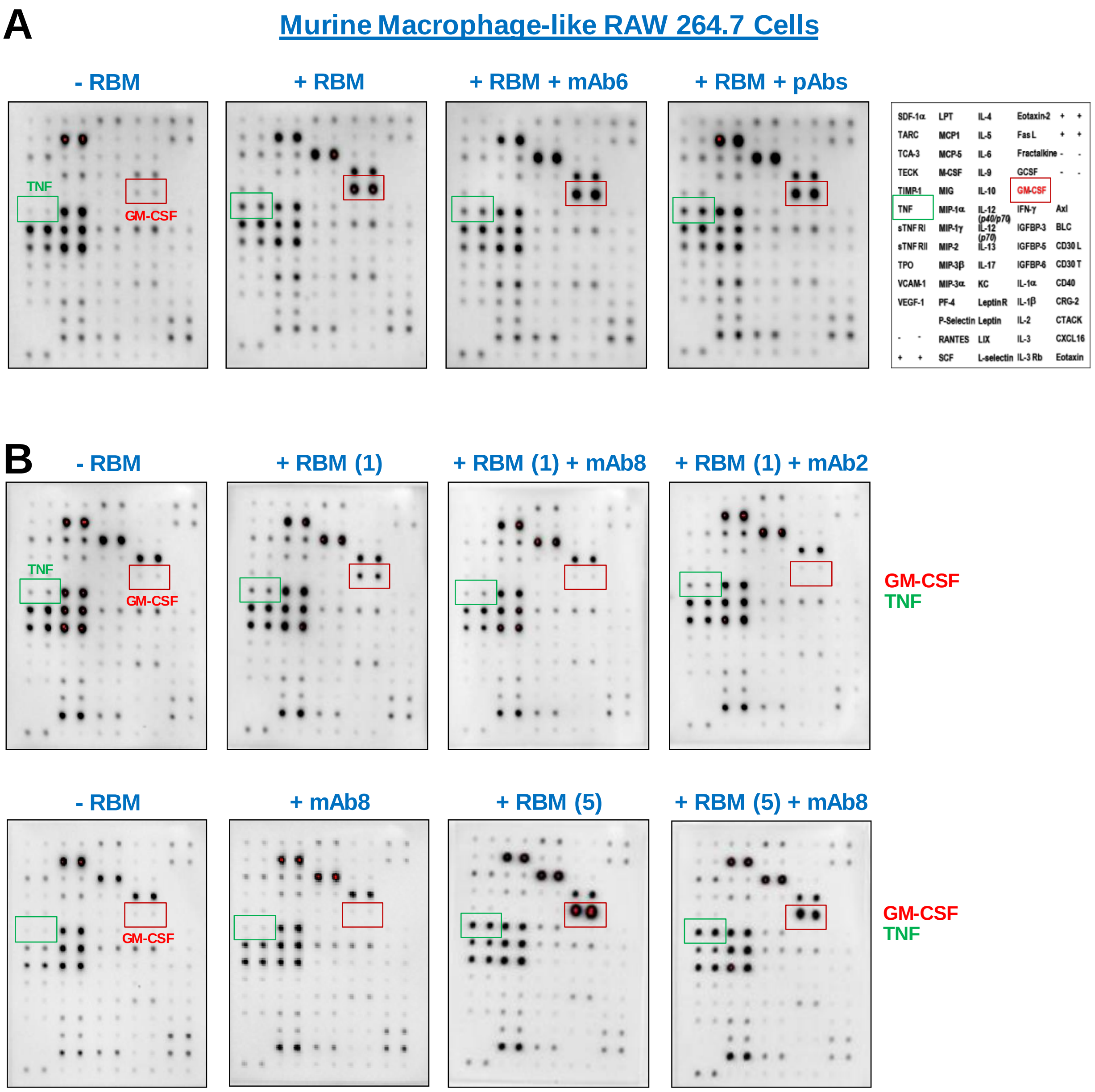


**Figure S3. RBM-reactive mAbs blocked the RBM-induced GM-CSF secretion in murine macrophage-like RAW 264.7 cells.** Murine macrophage-like RAW 264.7 cells were stimulated with recombinant RBM (1.0 or 5.0 μg/ml) either alone or in the presence of one polyclonal antibodies (pAbs) and three different mAbs (mAb6, mAb8 and mAb2; at a molar ratio of 1:6) or irrelevant murine polyclonal antibodies (pAbs, IgGs), and extracellular concentrations of 62 cytokines and chemokines were measured by Cytokine Antibody Arrays at 16 h post stimulation. Shown here were some representative murine Cytokine Antibody Arrays included in the quantitative bar graph of Figure 2B.
