# Supplementary material for: Monoclonal antibodies capable of binding SARS‐CoV‐2 spike protein receptor‐binding motif specifically prevent GM‐CSF induction
Source: J Leukoc Biol. 2021 Mar 24;111(1):261–7. doi: 10.1002/JLB.3COVCRA0920-628RR (PMC8251270; doi:10.1002/JLB.3COVCRA0920-628RR)
Supplement: Supplementary file 4 — Figure S4. RBM induced GM‐CSF production in vivo. A) Western blotting analysis of lung ACE2 levels. [file JLB-111-261-s001.pdf]

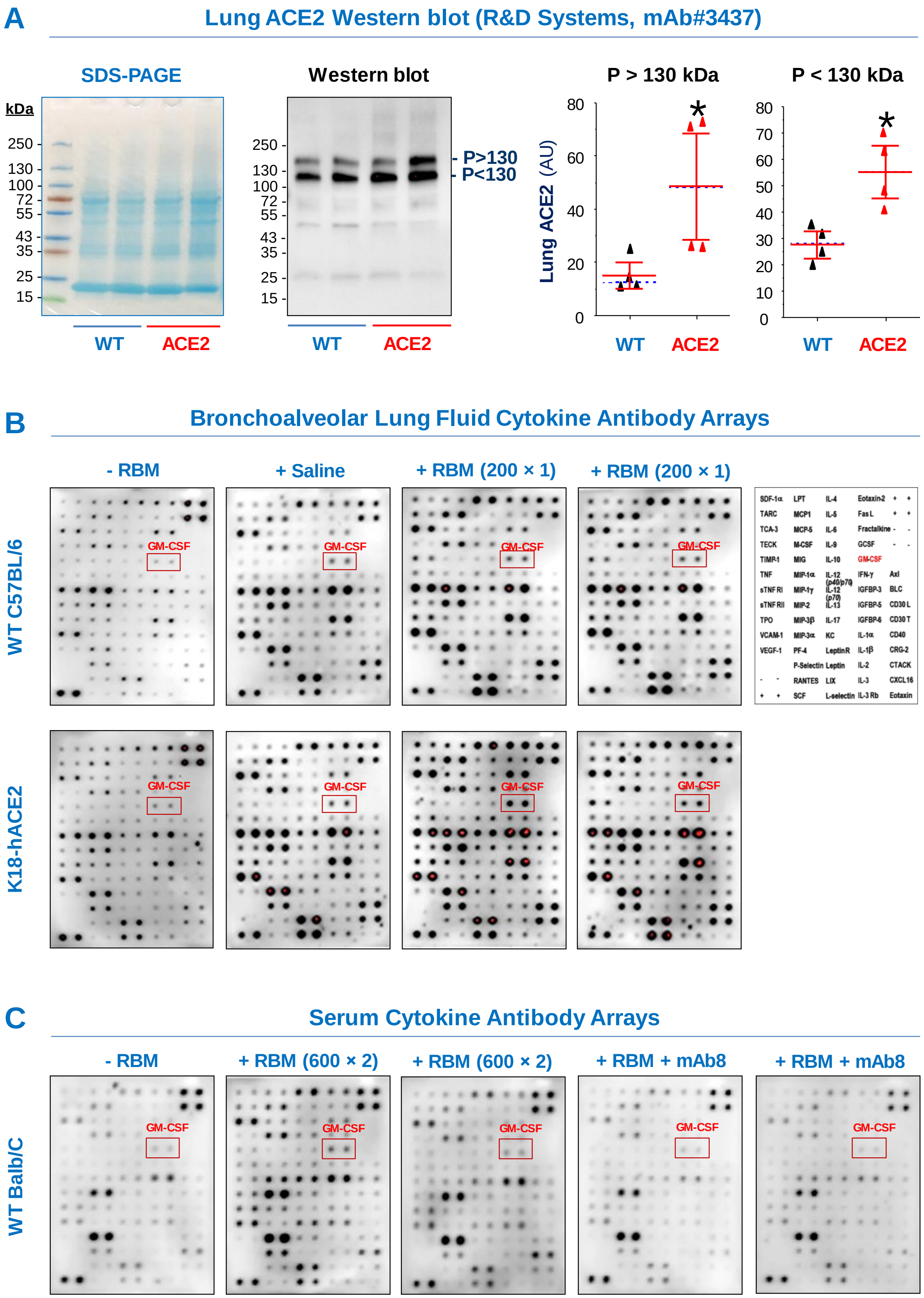
A
Lung ACE2 Western blot (R&D Systems, mAb#3437)
SDS-PAGE
kDa
250
130
100
72
55
43
35
25
15
WT
ACE2
Western blot
P>130
P<130
WT
ACE2
P > 130 kDa
P < 130 kDa
Lung ACE2 (AU)
*
*
WT
ACE2
WT
ACE2
B
Bronchoalveolar Lung Fluid Cytokine Antibody Arrays
- RBM
+ Saline
+ RBM (200 × 1)
+ RBM (200 × 1)
WT C57BL/6
K18-hACE2
GM-CSF
SDF-1α LPT IL-4 Eotaxin-2 + +
TARC MCP1 IL-5 Fas L + +
TCA-3 MCP-5 IL-6 Fractalkine - -
TECK M-CSF IL-9 GCSF - -
TIMP-1 MIG IL-10 GM-CSF
TNF MIP-1α IL-12 (p40/p70) IFN-γ Axl
sTNF RI MIP-1γ IL-12 (p70) IGFBP-3 BLC
sTNF RII MIP-2 IL-13 IGFBP-5 CD30 L
TPO MIP-3β IL-17 IGFBP-6 CD30 T
VCAM-1 MIP-3α KC IL-1α CD40
VEGF-1 PF-4 LeptinR IL-1β CRG-2
P-Selectin Leptin IL-2 CTACK
- - RANTES LIX IL-3 CXCL16
+ + SCF L-selectin IL-3 Rb Eotaxin
C
Serum Cytokine Antibody Arrays
- RBM
+ RBM (600 × 2)
+ RBM (600 × 2)
+ RBM + mAb8
+ RBM + mAb8
WT Balb/C
GM-CSF

Figure S4. RBM induced GM-CSF production *in vivo*. A) Western blotting analysis of lung ACE2 levels. Lung tissue were harvested from four wild-type C57BL/6 and four K18-hACE2 mice, and assayed for ACE2 levels by Western blotting analysis. Bar graph were relative band intensity of two reactive bands with molecular weights either slightly smaller (P <130 kDa) or greater (P >130 kDa) than 130 kDa, possibly indicative of distinct forms of ACE2 with different degree of post-translational modification (e.g., glycosylation) in murine lung tissues. *, $P < 0.05$ versus WT controls. **B) Intratracheal RBM administration elevated bronchoalveolar GM-CSF in transgenic K18-hACE2 mice over-expressing human ACE2**. Male wild-type C57BL/6 or transgenic mice over-expressing human ACE2 (K18-hACE2 mice) were intratracheally infused with saline once (100 µl) or saline containing RBM (200 µg). At 16 h post RBM administration, bronchoalveolar lung fluid was collected and subjected to Cytokine Antibody Assays. **C) RBM-reactive mAb8 attenuated the RBM-induced GM-CSF induction *in vivo*.** Male Balb/C mice were intraperitoneally administered with recombinant RBM twice at a higher dose (600 µg) either alone or in combination with a RBM-binding mAb (mAb8, 2.0 mg) at the same time. At 16 h post the initial RBM administration, animals were euthanized to harvest blood to measure serum levels of cytokines and chemokines using Cytokine Antibody Arrays. Shown here were some representative murine Cytokine Antibody Arrays included in the quantitative bar graph of **Figure 2C** and **Figure 2D**.
