# Supplementary material for: Monoclonal antibodies capable of binding SARS‐CoV‐2 spike protein receptor‐binding motif specifically prevent GM‐CSF induction
Source: J Leukoc Biol. 2021 Mar 24;111(1):261–7. doi: 10.1002/JLB.3COVCRA0920-628RR (PMC8251270; doi:10.1002/JLB.3COVCRA0920-628RR)
Supplement: Supplementary file 5 — Figure S5. Proposed model for mAb‐mediated inhibition of SARS‐CoV‐2 RBM‐induced GM‐CSF secretion. [file JLB-111-261-s004.pdf]

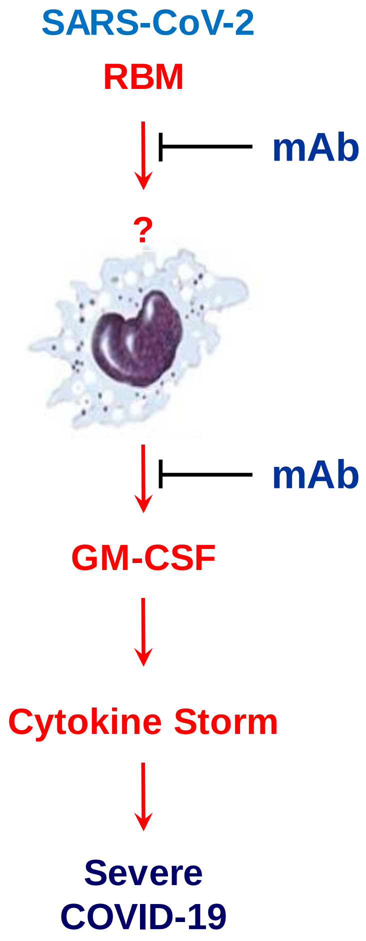

Figure S5. Proposed model for mAb-mediated inhibition of SARS-CoV-2 RBM-induced GM-CSF secretion.

SARS-CoV-2 RBM markedly induced GM-CSF secretion in human peripheral blood mononuclear cells (hPBMCs) and murine macrophage-like RAW 264.7 cells. Monoclonal antibodies capable of interrupting RBM-ACE2 interaction selectively impairs the RBM-induced GM-CSF secretion without affecting the RBM-induced release of other pro- (e.g., IL-1β, IL-6, TNF) and anti-inflammatory cytokines (e.g., IL-10) or chemokines (MCP-1 and MIP-1δ). Our important findings not only fully support the emerging notion that GM-CSF might be a key feature of SARS-CoV-2-induced cytokine storm in COVID-19 patients, but also suggests an exciting possibility to attenuate the SARS-CoV-2-induced GM-CSF production and "cytokine storm" to fight against severe COVID-19 using vaccines capable of eliciting RBM-targeting antibodies.
