# Supplementary material for: Monoclonal antibodies capable of binding SARS‐CoV‐2 spike protein receptor‐binding motif specifically prevent GM‐CSF induction
Source: J Leukoc Biol. 2021 Mar 24;111(1):261–7. doi: 10.1002/JLB.3COVCRA0920-628RR (PMC8251270; doi:10.1002/JLB.3COVCRA0920-628RR)
Supplement: Supplementary file 6 — Supplementary Material [file JLB-111-261-s003.docx]

**Supplemental Information**

**Extended Method**

**Material**

Murine macrophage-like RAW 264.7 cells were obtained from American Type Culture Collection (ATCC, Rockville, MD). Dulbecco’s modified Eagle medium (DMEM, 11995-065) and penicillin / streptomycin (Cat. #15140-122) were from Invitrogen/Life Technologies (Carlsbad, CA). Fetal bovine serum was from Crystalgen (FBS-500, Commack, NY) and heat-inactivated before use. The monoclonal antibodies against human tetranectin were generated in Balb/C and C57BL/6 mice at the GenScript (Piscataway, NJ, USA) as previously described ^18^. Irrelevant polyclonal antibodies (pAbs, IgGs) from non-immunized mice were purchased from the Sigma-Aldrich (Cat. #I5381). Several ACE2-reactive antibodies were obtained from the R&D Systems (rat mAb, Cat. #3437, Minneapolis, MN) and the Abcam (rabbit pAb, Cat. #ab87436; Cambridge, MA). Highly purified recombinant human ACE2 corresponding to the extracellular domain (Gln18-Ser740) was obtained from two different commercial sources, Biolegend (Cat. # 7920008) and Raybiotech (Cat. # 230-30165).

***Cell culture***

Human blood was purchased from the New York Blood Center (Long Island City, NY, USA), and human peripheral blood mononuclear cells (hPBMCs) were isolated by density gradient centrifugation through Ficoll (Ficoll-Paque PLUS) as previously described ^18^. Murine macrophage-like RAW 264.7 cells or human PBMCs (hPBMCs) were cultured in DMEM supplemented with 1% penicillin/streptomycin and 10% FBS (for ARW 264,7 cells) or 10% human serum (for hPBMCs). When they reached 70-80% confluence, adherent cells were gently washed with, and immediately cultured in, OPTI-MEM I before stimulating with highly purified recombinant RBD or RBM in the absence or presence of anti-TN mAbs. The extracellular concentrations of various cytokines/chemokines were determined by Cytokine Antibody Arrays as previously described ^41^.

***Preparation of recombinant RBD and RBM proteins***

The cDNAs encoding for the ACE2 receptor binding domain (RBD, residue 319 - 541) or receptor binding motif (RBM, residue 437-508) of SARS-CoV-2 spike protein (S) were cloned into a pCAL-n vector, and the recombinant proteins with an N-terminal Histidine Tag (6 × His) were expressed in E. coli BL21 (DE3) cells in the presence of 3.0 mM IPTG (isopropyl-1-thio-beta-D-galactopyranoside). Recombinant RBD and RBM proteins were isolated from the inclusion bodies by differential centrifugation, and further purified by urea (8.0 M Urea, 20 mM Tris-HCl, pH 8.9) solubilization and agarose bead-immobilized metal (Ni^2+^) affinity chromatography. After extensive washing with buffer 1 (20 mM Tris-HCl, 10 mM imidazole, 0.5 M NaCl, 8.0 M Urea, pH 8.0) and buffer 2 (20% DPBS1X, 10% glycerol, 8.0 M Urea, pH 7.5), the recombinant histidine-tagged RBD or RBM proteins were eluted with buffer containing 0.5 M Imidazole,10% Glycerol, 20% DPBS1X, 8.0 M Urea, pH 8.0. The recombinant proteins were then further purified by dialysis at 4⁰ C in buffer containing 20% DPBS1X, 10 % Glycerol and 0.5 mM TCEP, pH8.0. Recombinant proteins were tested for LPS content by the chromogenic *Limulus* amebocyte lysate assay (Endochrome; Charles River), and the endotoxin content was less than 0.01 U per microgram of recombinant proteins.

***Open Surface Plasmon Resonance (SPR)***

We used the Nicoya Lifesciences gold-nanoparticle-based Open Surface Plasmon Resonance (OpenSPR) technology to estimate the binding kinetics and affinity of ACE2 or monoclonal antibodies to SARS-CoV-2 RBD or RBM following the manufacturer’s instructions. For instance, highly purified recombinant RBD or RBM was immobilized on the NTA sensor chip (Cat. # SEN-Au-100-10-NTA), and ACE2 or mAb was applied at different concentrations. The response units were recorded over time, and the binding affinity was estimated as the equilibrium dissociation constant K_D_ using the Trace Drawer Kinetic Data Analysis v.1.6.1. (Nicoya Lifesciences) as previously described ^18^. To determine the possible competition with the human ACE2, SARS-CoV-2 RBM was immobilized to NTA sensor chips via histidine tag for a final RU around 500. A RBM-binding mAb was injected onto the chip until binding steady-state was reached, and ACE2 was re-injected as analyte at identical concentrations. The competition capacity of RBM-binding mAb was determined by the level of reduction in response units of ACE2 with and without prior mAb incubation. Results presented are representatives of two independent experiments.

***Cytokine Antibody Array***

Human Cytokine Antibody C3 Arrays (Cat. No. AAH-CYT-3-4, RayBiotech Inc), which detect 42 cytokines on one membrane, were used to determine cytokine concentrations in human PBMC-conditioned culture medium as previously described ^18^. Murine Cytokine Antibody Arrays (Cat. No. M0308003, RayBiotech Inc.), which simultaneously detect 62 cytokines on one membrane, were used to measure relative cytokine concentrations in macrophage-conditioned culture medium as described previously ^18;42^.

***Western blotting***

The concentrations of TN or ACE2 in lung tissue or murine macrophage-like RAW 264.7 cell lysate were determined by Western blotting analysis using homemade murine mAbs against human TN (mAb8, mAb2 or mAb6) or commercial antibodies against mouse ACE2 (residue 18-740, rat mAb, Cat. #3437, R&D Systems), human ACE2 (residue 18-237, mouse mAb, Cat. #15983, the Cell Signaling Technology) or human ACE2 (residue 350-450, rabbit pAb, Cat. #ab87436, Abcam). All these human ACE2-reactive antibodies were predicted to react with murine ACE2 as well. After blocking with 5% nonfat milk, the membranes were incubated with the appropriate antibodies (anti-TN, 1:1000; anti-ACE2, 1:500) overnight. Subsequently, the membranes were incubated with the appropriate secondary antibodies, and the immune-reactive bands were visualized by chemiluminescence. The relative band intensity was quantified using the UN-SCAN-IT Gel Analysis Software Version 7.1 (Silk Scientific Inc.).

***Animal experiments***

Wild-type Balb/C (Stock # 000651), wild-type C57BL/6 (Stock #000664), and transgenic mice over-expressing human ACE2 in epithelial cells (K18-hACE2 mice, Stock # 034860) were obtained from the Jackson Laboratory (Bar Harbor, ME), and acclimated for 1 week before use. This study was approved by the Feinstein Institutes for Medical Research Institutional Animal Care and Use Committee (IACUC) under Protocol #2016-028 (Term II, Modification #8, Approved on September 2, 2020) and #2017-027 (Term II, Modification #1, Approved on September 30, 2020). To assess the GM-CSF-induction property of RBM *in vivo*, wild-type C57BL/6 (male, 7-8 weeks old, 20-25 g) and K18-hACE2 (male, 7-8 weeks old, 20-25 g) mice were anesthetized under isoflurane, and intratracheally administered with saline (1 × PBS, 100 µl) or equal volume of saline containing recombinant RBM (200 µg/mouse). At 24 h post RBM administration, animals were euthanized by CO_2_ asphyxiation to harvest bronchoalveolar lung fluid (BALF) by repetitively lavaging the lungs with 1.0 ml T-PER Tissue Protein Extraction Reagent (Cat. #78510, Thermofisher Scientific, Waltham, MA) containing protease inhibitors (Cat. #A32953, Thermofisher Scientific) for a total of three times. Equal volume of BALF samples were then subjected to Cytokine Antibody Array analysis. To evaluate the RBM-inhibiting capacity of mAb8, Balb/C (male, 7-8 weeks, 20-25 g) mice were intraperitoneally and repetitively (at t = 0 and t = 12 h) administered with recombinant RBM (600 µg/mouse, 100 µl) either alone or in combination with mAb8 (2.0 mg/mouse, intraperitoneally, twice at t = 0 and t = 12 h). At 16 h post the initial RBM administration, animals were euthanized to harvest blood to measure serum levels of cytokines and chemokines using Cytokine Antibody Arrays.

***Statistical analysis***

All data were assessed for normality by the Shapiro-Wilk test before conducting statistical tests among multiple groups by one-way analyses of variance (ANOVA) followed by the Fisher Least Significant Difference (LSD) test. A *P* value < 0.05 was considered statistically significant.

**References**

41. Chen W, Zhu S, Wang Y et al. Enhanced Macrophage Pannexin 1 Expression and Hemichannel Activation Exacerbates Lethal Experimental Sepsis. *Sci Rep* 2019; 9(1):160-37232.

42. Li W, Bao G, Chen W et al. Connexin 43 Hemichannel as a Novel Mediator of Sterile and Infectious Inflammatory Diseases. *Sci Rep* 2018; 8(1):166-18452.
